# Influenza Risk Management: Lessons Learned from an A(H1N1) pdm09 Outbreak Investigation in an Operational Military Setting

**DOI:** 10.1371/journal.pone.0068639

**Published:** 2013-07-10

**Authors:** Margaret Farrell, Peter Sebeny, John D. Klena, Cecilia DeMattos, Guillermo Pimentel, Mark Turner, Antony Joseph, Jennifer Espiritu, John Zumwalt, Erica Dueger

**Affiliations:** 1 Global Disease Detection and Response Program, United States of America. Naval Medical Research Unit No, Cairo, Egypt; 2 Clinical Trials and Military Studies Department, United States Naval Medical Research Unit No. 3, Cairo, Egypt; 3 Naval Health Research Center, San Diego, California, United States of America; 4 Viral and Zoonotic Disease Research Program, United States Naval Medical Research Unit No. 3, Cairo, Egypt; 5 Naval Hospital Bremerton, Bremerton, Washington, United States of America; 6 Navy Environmental and Preventative Medicine Unit, Naval Medical Center San Diego, San Diego, California, United States of America; 7 Epidemic Intelligence Service Program, United States Centers for Disease Control and Prevention, Atlanta, Georgia, United States of America; 8 Navy Hospital Jacksonville, Naval Air Station Jacksonville, Jacksonville, Florida, United States of America; 9 Global Disease Detection Branch, United States Centers for Disease Control and Prevention, Atlanta, Georgia, United States of America; National Center for Biotechnology Information (NCBI), United States of America

## Abstract

**Background:**

At the onset of an influenza pandemic, when the severity of a novel strain is still undetermined and there is a threat of introduction into a new environment, e.g., via the deployment of military troops, sensitive screening criteria and conservative isolation practices are generally recommended.

**Objectives:**

In response to elevated rates of influenza-like illness among U.S. military base camps in Kuwait, U.S. Naval Medical Research Unit No. 3 partnered with local U.S. Army medical units to conduct an A(H1N1) pdm09 outbreak investigation.

**Patients/Methods:**

Initial clinical data and nasal specimens were collected via the existent passive surveillance system and active surveillance was conducted using a modified version of the World Health Organization/U.S. Centers for Disease Control and Prevention influenza-like illness case definition [fever (T > 100.5˚F/38˚C) in addition to cough and/or sore throat in the previous 72 hours] as the screening criteria. Samples were tested via real-time reverse-transcription PCR and sequenced for comparison to global A(H1N1) pdm09 viruses from the same time period.

**Results:**

The screening criteria used in Kuwait proved insensitive, capturing only 16% of A(H1N1) pdm09-positive individuals. While still not ideal, using cough as the sole screening criteria would have increased sensitivity to 73%.

**Conclusions:**

The results of and lessons learned from this outbreak investigation suggest that pandemic influenza risk management should be a dynamic process (as information becomes available regarding true attack rates and associated mortality, screening and isolation criteria should be re-evaluated and revised as appropriate), and that military operational environments present unique challenges to influenza surveillance.

## Introduction

Respiratory illnesses, including influenza, constitute a significant disease burden in military settings. Historically, deployed military troops are at increased risk of respiratory disease due to high attack rates, rapid onset and difficulty controlling transmission in austere and crowded environments [1,2]. As demonstrated in the influenza pandemics of 1918 and 1919, transmission of influenza among service members can play an important role in transcontinental spread of influenza. In fact, the first documented influenza outbreak in the spring of 1918, prior to the pandemic, was among recruits at Fort Riley, Kansas [3]. As recently as the 2009 H1N1 pandemic, the first evidence of community transmission of the virus in Spain was detected during an outbreak investigation of influenza-like illness (ILI) in soldiers from an engineering military academy [4].

The United States (U.S.) Department of Defense (DOD) influenza surveillance program is two-pronged, with one focusing on DOD active duty members and their beneficiaries and another focusing on local capacity building to improve surveillance in strategic areas of the world. The Armed Forces Health Surveillance Center funds influenza surveillance programs in multiple locations, including overseas military bases [5]. Standardized epidemiologic data and a nasal wash are collected from individuals who meet the World Health Organization (WHO) ILI case definition. Samples are transported to the U.S. Air Force School of Aerospace Medicine laboratories for influenza testing, subtyping, molecular characterization and analysis [5,6]. The DOD played an active role in the A(H1N1) pdm09 pandemic, including the detection of the first case in the U.S. [7]. Following the WHO declaration of a phase five pandemic alert on 29 April, 2009, DOD medical entities overseas were on alert for increased influenza-like activity.

In late April 2009, unusually high rates of ILI were detected by the existing passive surveillance system among U.S. military base camps in Kuwait. The ILI cases were concentrated at Camp Buehring, a U.S. Army base camp in northwest Kuwait that has served as a staging and training base for tens of thousands of U.S. troops deploying to Iraq and Afghanistan. U.S. Naval Medical Research Unit No. 3 (NAMRU-3), a DOD infectious disease research laboratory in Cairo, Egypt, was asked to provide influenza diagnostics and conduct an ILI outbreak investigation at Camp Buehring. This report describes several aspects of the investigation, including early active screening efforts and attempts to mitigate spread of ILI within the camp; , the genetic characteristics of the collected samples; and the challenges with and lessons learned from outbreak activities in an operational environment.

## Materials and Methods

### Recruitment and Enrollment

On 7 May 2009, NAMRU-3 began an outbreak investigation in collaboration with the Forward Deployed Medical Unit (FDPMU) assigned to Camp Arifjan, Kuwait. Initial activities were aimed at providing local diagnostic support for 24 respiratory samples that had already been collected via passive surveillance. Per routine clinical management, these samples had been collected via nasal wash from patients who presented at U.S. military clinics throughout Kuwait, and were tested using the QuickVue influenza test (Quidel, San Diego, CA, USA), a rapid influenza antigen test. These samples were subsequently processed for RNA and analyzed by real-time reverse transcriptase PCR (rt-RT-PCR) at Camp Arifjan using a Light Cycler 2.0 thermocycler (Roche, Indianapolis, IN, USA) to rule out seasonal influenza. As the majority of the initial cases were identified at the Camp Buehring Troop Medical Clinic (TMC) following the first week of testing, the thermocycler and related supplies were relocated to Camp Buehring, where they remained for the duration of the outbreak. All 24 specimens from the initial batch were transported to NAMRU-3 for confirmatory testing via the Applied Biosystems 7500 Fast PCR system (Applied Biosytems, Foster City, CA, USA).

Additionally, on 19 May, the NAMRU-3 investigation team, in collaboration with the Camp Buehring medical staff, conducted an active influenza screening for A(H1N1) pdm09 among two U.S. Army units that had recently arrived at Camp Buehring. The screening included 217 troops representing the 659^th^ Maintenance Company from Fort Bragg, North Carolina (arrived on 7 May) and F Troop 3-227 from Fort Hood, Texas (arrived on 30 April). The outbreak screening criteria were a modified version of the WHO/US Centers for Disease Control and Prevention (CDC) ILI case definition [fever (T > 100.5˚F/38˚C) in addition to cough and/or sore throat in the previous 72 hours]. History/unknown history of fever was added to these screening criteria due to the known high prevalence of self treatment with anti-pyretics within mobilizing military units.

A nasopharyngeal (NP) swab was collected, body temperature was recorded, and the soldiers self-reported their symptoms via a standardized questionnaire. Specimens were collected from all soldiers, regardless of reported symptoms. Soldiers who met the screening criteria were sent to the Camp Buehring TMC for further evaluation and possible quarantine. All of the associated NP swabs were transferred to NAMRU-3 for advanced testing. Laboratory results from NAMRU-3 for both the initial batch and the screening samples were linked using unique identifiers. Data were double-entered into a Microsoft Access database and analyzed using Microsoft Excel and SPSS 19.0 statistical software (SPSS Inc., Chicago, IL, USA).

Reported Data was collected as part of an outbreak investigation, supporting International Health Regulation (IHR) required reporting of a Public Health Event of International Concern (PHEIC). At the time of data collection, U.S. Naval Medical Research Unit No. 3 (NAMRU-3) Institutional Review Board classified all outbreak investigations as activities that do not involve human research and therefore do not require participant consent. The outbreak investigation was conducted on a U.S military base in Kuwait under the authority of the base commanding officer; consultation with the local authorities was not required. Participants voluntarily agreed to provide a naso-pharyngeal swab and to complete an outbreak investigation questionnaire. While personally identifying information was initially collected in conjunction with the screening process in order to facilitate the isolation of soldiers with suspected infection, the database containing this information was de-identified prior to analysis. The NAMRU-3 Institutional Review Board approves the publication of the results of this outbreak investigation.

### RNA extraction

RNA was extracted from NP swabs in viral transport medium or nasal washes (140µL) using the QIAamp viral RNA mini extraction kit as recommended by the manufacturer (Qiagen, Valencia, CA, USA).

### Real-time reverse-transcription polymerase chain reaction (rt-RT-PCR)

In Kuwait, cDNA production and specific amplification of each target gene was conducted in one tube, using the Superscript III RT/Platinum Taq mix (Invitrogen, Carlsbad, CA, USA) and all reactions were executed using a LightCycler 2.0 (Roche). Influenza testing was performed using the three-target detection system (universal influenza A via the matrix gene, seasonal H1 and seasonal H3) developed by CDC, Atlanta [8]. The human RNase P gene was used as a positive control. For all rt-RT-PCR assays a negative (no template) control and positive template control were included for each primer set as per the established protocols. At NAMRU-3, in addition to confirmation of the Kuwait rt-RT-PCR results, RNA samples were assayed for the presence of A(H1N1) pdm09 (using the SwInfA primer and probe set) and specific A(H1N1) pdm09 influenza A viruses using SwH1 primer and probe set developed by CDC, Atlanta (WHO Collaborating Center for Influenza at CDC-Atlanta 2009; CDC protocol of rt-RT-PCR for A(H1N1) pdm09, Atlanta, GA, USA). Assays were performed using an ABI 7500 DX Fast (Applied Biosystems).

### DNA sequence analysis

Sequence analysis was performed on PCR products obtained after reverse transcription and amplification of RNA extracted from viral cultures. Complete nucleotide coding sequences of the hemagglutinin (HA) gene were determined by cycle sequencing [9]. The Kuwait nucleotide sequences were aligned with 1047 A(H1N1) pdm09 HA genes (USA, 558; rest of the Americas, 190; Oceania, 5; Europe, 191; Asia, 103) from samples collected between 1 April and 31 May 2009 and obtained from GenBank. Phylogenetic analyses were carried out using the programs Clustal W/X (Version 2.1), DNASTAR Lasergene MegAlign (Version 7.1.0, DNASTAR Software for Life Sciences, Madison, WI, USA) and MEGA 5.05 [10,11]. A maximum likelihood (ML) dendrogram using the Kimura-2 parameter model with 1000 bootstrap replicates was constructed and the nearest neighbor interchange for the ML heuristic method was used for tree inference. The genbank accession numbers for the Kuwait HA gene sequences reported in this paper are CY062373, CY062374, CY062375 and CY062376.

### Viral culture

Influenza viruses were isolated from clinical samples by infecting Madin Darby Canine Kidney (MDCK) (ATCC CCL-34) cells following standard cell culture and virus isolation [12,13]. The specimens were treated with 5 mg/ml gentamicin (GIBCO, Carlsbad, CA, USA), 1000 U/ml penicillin (GIBCO), 1000µg/ml streptomycin (GIBCO) and 25 µg/ml amphotericin B (GIBCO) prior to inoculation. An MDCK cell tube was inoculated with 100µl of the treated samples, incubated at 37^°^C in a 5% CO_2_ atmosphere and monitored daily for the appearance of cytopathic effects (CPE) for 10 days. All cultures were screened by hemagglutination to detect viral presence. If no CPE or hemagglutination at day 10 was observed, a second passage of the specimens was performed. Viral identification was carried out by rt-RT-PCR and sequence analysis. The obtained isolates were stored at -70^°^C for further analyses.

## Results

Of the initial 24 specimens transported to NAMRU-3 for confirmatory testing, 18 (75%) were PCR positive for A(H1N1) pdm09. Of the 217 NP specimens collected during the active screening on 19 May, 44 (20%) were positive for A(H1N1) pdm09 and 173 (80%) were influenza A negative. At the time of the screening, the soldiers had been in Kuwait for an average length of 14 days (Range: 6–19 days), and their symptoms were as follows: 15 (7%) of the soldiers self-reported a history of fever within 72 hours, 77 (35%) self-reported a cough, 43 (20%) self-reported a sore throat and current fever was detected in two (<1%) soldiers. The positive predictive value (PPV), negative predictive value (NPV), sensitivity and specificity of the modified screening criteria used at the 19 May screening in Camp Buehring (including the addition of history/unknown history of fever) were calculated and compared with the more specific standard WHO ILI screening criteria, as well as a more sensitive sole criterion of cough within 72 hours. Table 1 provides the details of these comparisons, and a supplementary raw data file contains the associated data.

**Table 1:** A comparison of screening criteria based on results of the 19 May, 2009 active screening, Camp Buehring.

**Screening Criteria**	**PPV**	**NPV**	**Sensitivity**	**Specificity**
Cough alone	42	91	73	74
Fever/hx of fever/UNK hx of fever + (cough or sore throat)	32	81	16	91
Current fever + (cough or sore throat)	100	80	5	100

Complete HA nucleotide sequence was obtained for four cultured isolates obtained from the 18 A(H1N1) pdm09 PCR-positive clinical samples received by NAMRU-3. The Kuwait HA genes were 100% identical to each other in their nucleotide sequence (henceforth referred to as the Kuwait HA gene). The Kuwait HA gene was compared with 558 HA sequences of A(H1N1) pdm09 viruses circulating in the U.S. between 1 April and 31 May 2009 (data not shown). Despite the high degree of genetic homology within the U.S. group (99.8% average intrinsic homology), the Kuwaiti samples segregated in a separate clade that was statistically supported (73% bootstrap value). The Kuwait HA gene contained two mutations at nucleotide positions 687 and 1029 that were not translated into amino acid changes (silent mutations) and were not present in the U.S. viruses. The translated Kuwait HA protein showed 100% amino acid identity with the HA proteins of some of the viruses present throughout the U.S. (Arizona, Illinois, Iowa, New Mexico, New York, North Dakota, Oklahoma, Oregon, Texas, Washington and Wisconsin) indicating that influenza viruses carrying an HA protein identical to the Kuwait samples were already circulating in this country during this time period.

**Figure 1:**
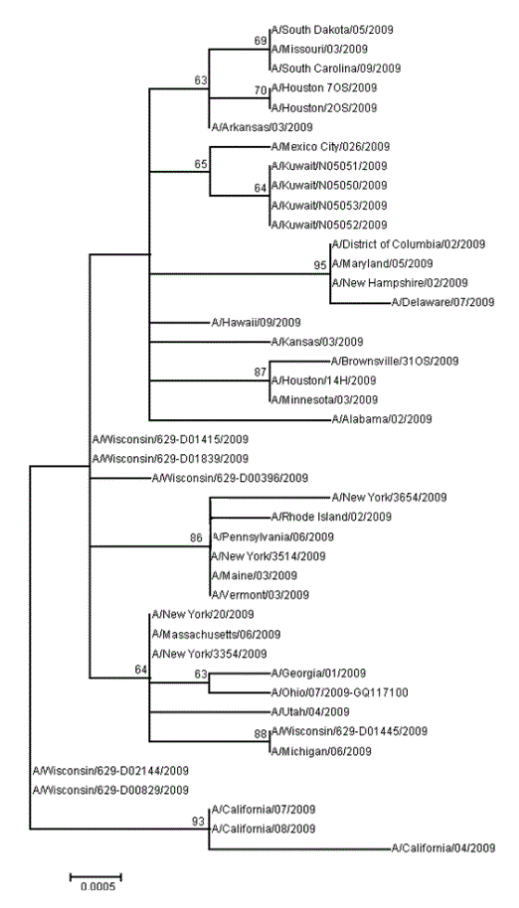
A phylogenetic tree of A(H1N1) pdm09 nucleotide sequences.

When the Kuwait HA gene sequence was compared to nucleotide sequences from A(H1N1) pdm09 viruses collected worldwide during the same time period, it was shown that only one sample from Mexico City shared the same silent nucleotide change at position 1029 forming a branch close to the Kuwait HA gene group (Figure 1).

This phylogenetic tree of A(H1N1) pdm09 nucleotide sequences is representative of viruses circulating in the U.S. between 01 April and 31 May, 2009, the A/Mexico City/026/2009 virus and the Kuwait viruses isolated during the Camp Buhering outbreak. The analysis was carried out using the Maximum Likelihood method of the MEGA 5.05 software with 1,000 replicates. Only bootstrap values higher than 60% are shown. The strain names are indicated in the tree. The GenBank accession numbers for the Kuwait isolates are CY062373 to CY062376. The remaining sequences were obtained from GenBank.

When the Kuwait HA translated protein was compared with the amino acid sequences from A(H1N1) pdm09 from other regions of the world, it showed 100% amino acid identity with viruses from Europe (Belgium, France, Germany, Italy, Malta, Norway and the United Kingdom), Asia (China and Japan), Oceania (New Zealand) and North, Central and South America (Brazil, Canada, Colombia, Costa Rica, El Salvador, Mexico and Panama). This result indicates that subpopulations of influenza viruses carrying an HA amino acid sequence identical to the Kuwait protein were co-circulating globally at the time of the Kuwait outbreak.

## Discussion

The outbreak investigation at Camp Buehring led to the first reported cases of A(H1N1) pdm09 in Kuwait, and the laboratory results from the 19 May screening confirmed high rates of current infection with A(H1N1) pdm09 and ILI in at least two recently arrived U.S. military units. The introduction of the virus into Kuwait raised significant geopolitical concerns early in the pandemic when the severity of A(H1N1) pdm09 was still unclear.

Since deployed military troops represent a population who may be especially susceptible to and capable of viral transmission, operational military preventive medicine has traditionally focused on preventing soldiers from acquiring infections abroad and transporting agents to the U.S. In addition to internal public health risk management, however, the DOD has the added medical and political responsibility of minimizing the introduction of influenza into international areas of deployment. During the outbreak in Kuwait, there was significant potential for spread of the A(H1N1) pdm091strain from U.S. Army installations to various parts of the world via deployments and regional troop movement. For example, there are many Kuwaiti national contractors who work on the military camps in Kuwait, but live off-base, and many U.S. service members who were training in Kuwait, were deployed to Afghanistan and Iraq where A(H1N1) pdm09 virus was later noted among U.S. service members in large numbers [14].

Active screening for ILI in operational military settings during a pandemic is also critical because the physical strain and fatigue of general flu symptoms may impact a soldier’s ability to maintain situational awareness and top performance in the field. Deployed service members operate in harsh and dynamic environments where they are constantly contending with stressors associated with an impending deployment, lengthy workdays, rigorous training schedules, severe physical demands and extreme temperatures. Lastly, when soldiers are not actively training, they are billeted en masse in tents and trailers where viral transmission is unavoidable. Even an outbreak of mild influenza has the potential to render both individual soldiers and whole units unfit for deployment.

The A(H1N1) pdm09 pandemic prompted questions regarding the best methods and timing for screening of U.S. military personnel in order to reduce the risk of influenza transmission in-theater. For general, passive influenza surveillance, when establishing screening and isolation criteria in a U.S. military environment where troops have easy access to and often overuse non-steroidal medications, military medical leadership might consider expanding the standard ILI screening criteria to include a history of fever. Additionally, due to deployed soldiers’ generally strong sense of camaraderie and commitment to operational goals and timelines, leadership might also take into account that the use of self-reported symptoms may be ineffective. When faced with an influenza epidemic or pandemic in a military environment, however, flexibility is crucial to allow rapid transition from a passive to an active surveillance system. At the onset, when the severity of the strain is yet unknown and there is concern over introducing the strain into a new environment, the employment of more sensitive criteria for screening and potential isolation is crucial.

There are three major time points at which to consider the establishment of active screening of soldiers: (1) immediately before deployment from the U.S., (2) upon arrival at the interim training base, and (3) at the time of forward deployment to their ultimate duty station. At each of these time points, not only are these transient military personnel in a position to potentially introduce a virus into a new environment, but they are also amassed and readily accessible, i.e., logistically, it is an ideal opportunity to collect specimens and clinical data.

During the active screening at Camp Buehring, even the more sensitive, modified WHO ILI screening criteria failed to capture 84.0% of PCR-positive individuals (i.e., these soldiers were not recommended for isolation). Additionally, the 91.3% of influenza A negative soldiers who met the screening criteria may not have needed to be separated from their platoons. While both the standard WHO ILI and the modified WHO ILI screening criteria proved highly specific (100% and 91.3%, respectively), both screening criteria had extremely low sensitivities (4.5% and 15.9%, respectively), a key factor at this early stage in the pandemic. In an operational military setting in the face of an influenza pandemic of unknown severity, cough within 72 hours may be a simple, easily observed and effective screening criterion; using cough as the sole criterion during the Kuwait screening of recently arrived recruits would have increased the sensitivity to 72.7%. It is important to keep in mind, however, that the low specificity of cough alone, especially in a dusty desert environment, makes this sole screening criterion inappropriate for prospective passive screening.

At the onset of an influenza pandemic such as that of 2009, it is arguably appropriate to prioritize sensitivity over specificity until the severity of the virus can be evaluated. Military medical commands, however, should have systems in place to reevaluate and modify screening and isolation criteria as applicable when the severity of the strain becomes more apparent. As further information becomes available regarding the true attack rates and mortality associated with the novel pandemic virus, screening and isolation criteria should be re-evaluated and possibly revised to avoid the unnecessary isolation of uninfected soldiers, as appropriate.

The importance of the availability of reliable, local, real-time diagnostics is another key lesson learned from this investigation. Influenza samples from overseas DOD collection sites are generally shipped to the U.S., requiring maintenance of a cold chain for up to several weeks before arrival and testing. At the time of the initial outbreak in Kuwait, extensive confirmatory lab testing was underway to determine the scope of the pandemic within the U.S., and the existing system was not equipped to prioritize samples from regions that were initially considered to be free from pandemic influenza transmission. Additionally, at the onset of the A(H1N1) pdm09 pandemic, there was an over-reliance on the QuikVue test, which is known to be relatively insensitive, for on-site testing of nasal washes [7]. Diagnostic obstacles in the midst of a pandemic can significantly delay the reporting of pivotal screening results from deployed U.S. military personnel. The incorporation of a regional DOD laboratory, such as NAMRU-3, for confirmatory testing and outbreak assistance could enhance the quality and timely reporting time of laboratory data.

Since in-theater laboratories are accredited by the College of American Pathologists and approved for real-time PCR performance by the U.S. Food and Drug Administration (FDA), DOD could quickly introduce FDA-approved real-time PCR protocols, i.e., CDC seasonal influenza PCR protocols, to identify influenza A and detect the emergence of a novel strain. Within a few hours, results could be reported to the clinic collecting the samples where ILI cases could be entered into a local or online database, thus allowing cases to be tracked in real time and potentially expedite the identification of emerging epidemics.

A diagnostic alternative for combat-zone hospital laboratories where Applied Biosystems real-time PCR instruments are not available could be a rugged, portable real-time PCR platform, such as the Joint Biological Agent Identification and Diagnostic System (JBAIDS). Based on the demonstrated utility of JBAIDS during the Camp Buehring outbreak investigation as compared in parallel testing with the Lightcycler 2.0 platform (data not shown), NAMRU-3 recommends that CDC pursue validation of its seasonal diagnostic influenza assays and other outbreak-prone strains, e.g., avian influenza (H5N1) on this platform.

Exceptionally intensified epidemiologic surveillance and molecular characterization of A(H1N1) pdm09 viruses in the early stages of the 2009 pandemic allowed for the detection of genetic diversity among circulating viruses, the establishment of epidemiologic relationships between cases and outbreaks, and an enhanced understanding of influenza transmission, geographical distribution and virus evolution [15-19]. Some of this observed genetic diversity occurred in response to host adaptation, but over time, mildly deleterious mutations began to disappear from the circulating virus population [16]. The analyses of the Camp Buehring HA sequences with those from viruses from the Middle East, US, and Mexico obtained later in the pandemic showed that the synonymous Kuwaiti mutations at positions 697 and 1029 disappeared from circulating viral populations.

The main source of variation resulting in synonymous mutations is the viral replication frequency. Replication frequency depends on viral replication strategy and is highest for viruses that cause acute infections and are transmitted via aerosols. These conditions are achieved during epidemiologic situations where high rates of infection in crowded human populations are observed, such as those observed in military recruits in the U.S. and on the U.S. bases in Kuwait during the A(H1N1) pdm09 pandemic [20].

The HA gene from the Kuwait A(H1N1) pdm09 viruses contained two unique but synonymous mutations at the time of the outbreak at Camp Buerhing. One (nucleotide position 1029) was already circulating in Mexico. North America was the only identified geographical region in which this nucleotide change was present. Because this pandemic virus may cryptically circulate in an area for two to six weeks prior to detection, it is likely that the Kuwaiti A(H1N1) pdm09 virus was circulating among soldiers during their training in the U.S., but went undetected [17]. The molecular results support the findings of the epidemiologic investigation; however, the lack of U.S. sequences identical to the Kuwaiti samples precludes the pinpointing of the exact geographical area of virus origin within the U.S.

The results of this outbreak investigation demonstrate the importance of establishing sound, standardized passive influenza surveillance systems in operational military environments that can be readily converted into active surveillance in the event of a threat. While the current DOD system employs a standard ILI case definition and provides recommendations regarding minimum weekly sample collection, because there is no standardized method of collection, case clusters may go unrecognized. Additionally, the weekly summary of aggregate influenza cases captured via the DOD system does not incorporate data from overseas clinics like those in Kuwait.

The events of this pandemic also emphasize the importance of establishing a standardized process for the reporting of influenza events among U.S. troops in international areas of deployment. The Camp Buehring A(H1N1) pdm09 outbreak brought the potential for significant medical and political implications affecting U.S. military leadership, the government of Kuwait, and other U.S. Government and global agencies, e.g., CDC and WHO. Standardized reporting process should take into consideration the sensitivities and health policies of the host nation as well as international health regulations. In order to expedite the distribution of this potentially critical data to host nations, the U.S. military service branches should work with WHO to streamline processes regarding the reporting of infectious diseases among deployed troops.

Two significant limitations of the outbreak investigation at Camp Buehring were the inconsistency in sample collection and testing methods (nasal washes vs. NP swabs and QuickVue vs. PCR) and the limited population that were actively screened. Influenza risk management among deployed soldiers may be enhanced by standardizing screening, data/sample collection, and sample processing procedures. Additional suggestions for improved influenza risk management measures include standardizing treatment and isolation processes, promoting hand-washing, ensuring the availability of local real-time diagnostics, and incorporating data from international areas of deployment into DOD data reporting systems.

## References

[1] Balicer RD, Huerta M, Levy Y, Davidovitch N, Grotto I (2005) Influenza outbreak control in confined settings. Emerg Infect Dis 11(4): 579-583. doi:10.3201/eid1104.040845. PubMed: 15829197. 15829197 10.3201/eid1104.040845 PMC3320335

[2] Kiliç S, Gray GC (2007) Nonpharmaceutical Interventions for Military Populations During Pandemic Influenza. Turk Silahli Kuvvetleri Koruyucu Hekim Bul 6(4): 285-290. PubMed: 18516249. 18516249 PMC2405938

[3] Stone W, Swift G (1919) Influenza and influenzal pneumonia at Fort Riley, Kansas. JAMA 72(7): 487-493. doi:10.1001/jama.1919.02610070025014.

[4] Mayo Montero E, Ballester Orcal E, Piñeyroa Sierra AJ, Fé Marques A (2010) Santa-Olalla Peralta P, et al.. Pandemic Influenza (H 1 N 1) 2009 Outbreak in a Military Academy: start of community circulation in Spain. Rev Esp Salud Publica 84(5):597-607 10.1590/s1135-57272010000500011 21203722

[5] Kapp L, Jansen DJ (2009) The role of the Department of Defense during a flu pandemic. Washington (DC): CRS Report for Congress (US); June. 18 p Report No. R40619

[6] Committee for the Assessment of Dod-GEIS Influenza Surveillance and Response Programs (2007) Review of the DoD-GEIS influenza programs: strengthening global surveillance and response. Washington (DC): The National Academies Press.

[7] Faix DJ, Sherman SS, Waterman SH (2009) Rapid-test sensitivity for novel swine-origin influenza A (H1N1) virus in humans. N Engl J Med 361(7): 728-729. doi:10.1056/NEJMc0904264. PubMed: 19564634. 19564634 10.1056/NEJMc0904264

[8] Selvaraju SB, Selvarangan R (2010) Evaluation of three influenza A and B r e a l - t i m e r e v e r s e t r a n s c r i p t i o n - P C R a s s a y s a n d a n e w 2 0 0 9 H 1 N 1 a s s a y f o r d e t e c t i o n o f i n f l u e n z a v i r u s e s . J Clin Microbiol 48(11): 3870-3875. doi:10.1128/JCM.02464-09. PubMed: 20844230. 20844230 10.1128/JCM.02464-09 PMC3020824

[9] World Health Organization (2009) Sequencing primers and protocol. Available: http://www.who.int/csr/resources/publications/swineflu/GenomePrimers_20090512.pdf. Accessed: 23 December 2011.

[10] Larkin MA, Blackshields G, Brown NP, Chenna R, McGettigan PA et al. (2007) Clustal W and Clustal X version 2.0. Bioinformatics 23(21): 2947-2948. doi:10.1093/bioinformatics/btm404. PubMed: 17846036. 17846036 10.1093/bioinformatics/btm404

[11] Tamura K, Peterson D, Peterson N, Stecher G, Nei M et al. (2011). Molecular Evol Genet Anal Mega 5: 05 Available: http://www.megasoftware.net/mega.php. Accessed: 23 December 2011.

[12] World Health Organization (2002) WHO Manual on Animal Influenza Diagnosis and Surveillance [pamphlet].

[13] Szretter KJ, Balish AL, Katz JM (2006) Influenza: propagation, quantification, and storage. Curr Protoc Microbiol Chapter 15: Unit 15G.1:Unit 15G.1. PubMed : 18770580 10.1002/0471729256.mc15g01s3 18770580

[14] World Health Organization (2011) Iraq: Situation Report on Influenza A H1N1 Pandemic (as of 11 Nov 2009). Available: http://www.emro.who.int/iraq/pdf/h1n1_11_11_09.pdf. Accessed: 04 April 2010.

[15] Smith GJ, Vijaykrishna D, Bahl J, Lycett SJ, Worobey M et al. (2009) Origins and evolutionary genomics of the 2009 s w i n e - o r i g i n H 1 N 1 i n f l u e n z a A e p i d e m i c . Nature 459(7250): 1122-1125. doi:10.1038/nature08182. PubMed: 19516283. 19516283 10.1038/nature08182

[16] Valli MB, Meschi S, Selleri M, Zaccaro P, Ippolito G et al. (2010) Evolutionary pattern of pandemic influenza H1N1) 2009 virus in the late phases of the 2009 pandemic. PLoS Curr 2:RRN1149 10.1371/currents.RRN1149 PMC2832123 20228856

[17] Nelson M, Spiro D, Wentworth D, Beck E, Fan J et al. (2009) The early diversification of influenza A/H1N1pdm. PLOS Curr 1: RRN1126:RRN1126. PubMed : 20029664 10.1371/currents.RRN1126 PMC2773564 20029664

[18] Shiino T, Okabe N, Yasui Y, Sunagawa T, Ujike M et al. (2010) Molecular evolutionary analysis of the influenza A H1N1)pdm, May-September, 2009: temporal and spatial spreading profile of the viruses in Japan. PLoS One 5(6):e11057. 20548780 10.1371/journal.pone.0011057 PMC2883557

[19] Potdar VA, Chadha MS, Jadhav SM, Mullick J, Cherian SS et al. (2010) Genetic characterization of the influenza A pandemic H1N1) 2009 virus isolates from India. PLoS One 5(3):e9693. 20300625 10.1371/journal.pone.0009693 PMC2837743

[20] Hanada K, Suzuki Y, Gojobori T (2004) A large variation in the rates of synonymous substitution for RNA viruses and its relationship to a diversity of viral infection and transmission modes. Mol Biol Evol 21(6): 1074-1080. doi:10.1093/molbev/msh109. PubMed: 15014142. 15014142 10.1093/molbev/msh109 PMC7107514

